# Water Resistant Self-Extinguishing Low Frequency Soundproofing Polyvinylpyrrolidone Based Electrospun Blankets

**DOI:** 10.3390/polym11071205

**Published:** 2019-07-19

**Authors:** Jessica Passaro, Paolo Russo, Aurelio Bifulco, Maria Teresa De Martino, Veronica Granata, Bonaventura Vitolo, Gino Iannace, Antonio Vecchione, Francesco Marulo, Francesco Branda

**Affiliations:** 1 Department of Chemical Materials and Industrial Production Engineering (DICMaPI), University of Naples Federico II, P.le Tecchio 80, 80125 Naples, Italy; 2 Department of Industrial Engineering, Aerospace Division, University of Naples Federico II, Via Claudio 21, 80125 Naples, Italy; 3 Eindhoven University of Technology, P.O. Box 513, 5600 MB Eindhoven, The Netherlands; 4 Department of Physics, University of Salerno, Via Giovanni Paolo II 132, 84084 Fisciano (Salerno), Italy; 5 GEVEN S.p.A., Via Boscofangone, 80035 Nola, Italy; 6 Department of Architecture and Industrial Design, University of Campania Luigi Vanvitelli, Borgo San Lorenzo, 81031 Aversa (Caserta), Italy; 7 CNR-SPIN of Salerno, c/o Department of Physics, University of Salerno, Via Giovanni Paolo II 132, 84084 Fisciano (Salerno), Italy

**Keywords:** electrospinning, stöber synthesis, PVP/silica gel composite, acoustic properties, fire behavior, water resistance, thermal conductivity, porosity, flow resistivity

## Abstract

This paper shows that an eco-friendly electrospinning process allows us to produce water resistant sound absorbers with reduced thickness and excellent sound-absorption properties in the low and medium frequency range (250–1600 Hz) for which which human sensitivity is high and traditional materials struggle to match, that also pass the fire tests which are mandatory in many engineering areas. The structure and composition were studied through Scanning Electron Microscopy (SEM), Fourier Transform InfraRed (FTIR) Spectroscopy and ThermoGravimetric Analysis (TGA). The density, porosity and flow resistivity were measured. Preliminary investigation of the thermal conductivity through Differential Scanning Calorimetry (DSC) shows that they have perspectives also for thermal insulation. The experimental results indicate that the achievements are to be ascribed to the chemical nature of Polyvinylpyrrolidone (PVP). PVP is, in fact, a polymeric lactam with a side polar group that may be easily released by a thermooxidative process. The side polar groups allow for using ethanol for electrospinning than relying on a good dispersion of silica gel particles. The silica particles dimensionally stabilize the mats upon thermal treatments and confer water resistance while strongly contributing to the self-extinguishing property of the materials.

## 1. Introduction

In recent years, polymers electrospinning has become an internationally highly recognized method for the preparation of polymer nanofibers with a broad range of complex architectures [1,2,3,4,5]. It is specifically known for allowing us to produce micro/nanofibers ranging from 2 nm to several micrometers [1,2,4,5,6], in contrast to conventional processes of synthetic fiber forming, where continuous fibers ranging from 10 μm to 500 μm are produced.

Electrospinning is found many applications in medical areas such as tissue engineering and drug delivery [1,7,8,9,10] and in technical areas relying on nanofibers with specific photonic, electronic, photocatalytic and magnetic properties [1]. Recently, multifunctional air filtration membranes [11,12,13] were also successfully developed through electrospinning. In the field of structural materials, electrospun nanofibers with excellent thermal and mechanical properties were successfully obtained for use as reinforcements of polymers [14,15].

This paper deals with a very new application in the field of soundproofing materials. Because of the ability to produce very fine fibers easily, electrospinning has been recently proposed for the production of soundproofing materials [16,17,18]. The high specific surface area, 100 to 10000 times greater than that of the traditional acoustical fibrous materials, is expected, in fact, to give high sound absorption coefficients, which are due to the higher friction between the air molecules of sound waves and the electrospun fibers and/or rubbing of the fibers.

Interest in this topic is great because noise pollution is one of the most widespread problems in modern society and constitutes a real danger to human health [16,19,20,21]. Noise produces direct and cumulative adverse effects that impair health and that degrade residential, social, working, and learning environments with corresponding real (economic) and intangible (well-being) losses [22].

The production of soundproofing materials has increased in many areas, including buildings, aircraft and spacecraft, ground and marine transportation vehicles, and manufacturing facilities. In the transportation industries, the need to produce soundproofing materials remains a big problem to cope with [16,20,23,24,25,26]. The available soundproofing materials, in fact, do possess good sound absorption properties in the high frequency range but little in the low and medium frequency range (250–1600 Hz), for which human sensitivity is high [26].

Light polymeric soundproofing materials (density = 63 kg/m^3^) that are of interest for the transportation industry were recently fabricated through electrospinning [27,28] consisting of blankets of electrospun polyvinylpyrrolidone (PVP), with an average fiber diameter in the range 1–3 μm. The blankets were obtained by stacking disks of electrospun mats on each other. It was shown [27] that PVP samples may have better sound absorption properties in the lower frequency range than materials of the same mass that are usually traded in the field of civil engineering and better sound absorption properties than materials, of same thickness, that are usually traded in both the civil and aerospace engineering fields.

It is worth remembering that PVP does possess [29] a unique combination of properties, such as good solubility in both water and a range of organic solvents, a remarkable capacity to interact with a wide variety of organic and inorganic compounds, good biocompatibility, and non-toxicity to living tissues. For these reasons PVP has been widely used [29] in the biomedical field, the cosmetic and food industrial sectors, which closely affect human health. PVP has also been widely used as a medical additive or polymeric modifier. In fact, PVP is one of the most frequently investigated classes of materials for usage in medicine and in other applications interfacing with biological systems [1,2,3,4,5]. From a chemical point of view, PVP is a polymeric lactam. The amphiphilicity is due to the presence of a more highly polar amide group than non-polar methylene groups in both the backbone and in the side rings. The PVP’s good solubility in polar solvents allowed us to obtain blankets through electrospinning of its ethanol solutions, with obvious advantages in term of process eco-friendliness with respect to the use of insoluble polymers in polar solvents. The drawback is that the good solubility in water does strongly limit the applications of the blankets. This is an important issue. It is well known [12] that most of the organic solvents used in solvent electrospinning are toxic and harmful, and the solvent accumulation from solution evaporation during the electrospinning process causes serious environmental pollution, which is harmful to physical health.

To avoid all this, solvent free electrospinning techniques, which have been recently reviewed [12] may be applied. Alternatively, the electrospun polymers must be crosslinked. As an example, recently, poly(vinyl alcohol) (PVA) and konjac glucomannan (KGM) acqueous solutions with citric acid (CA) were electrospun to produce nanofiber membranes. Afterwards, the electrospun membranes were submitted to a thermal treatment that allowed esterification between the hydroxyl groups of PVA and KGM and the carboxyl groups of CA, which changed the water-soluble material into an insoluble one. Something similar was made in the case of the present paper. It is well known in fact that PVP may be cross-linked through thermal treatment. The problem is that the needed thermal treatment leads to a strong unacceptable samples shrinkages. So as described in the following, the problem was solved by adding sub-micrometer silica particles.

Recently PVP/silica composite fibers, 380 ± 100 nm in diameter, were obtained through electrospinning [30]. Newsome et al. demonstrated that proper heat treatment of these composite fibers at 200 °C crosslinked the polymer yielding solvent-resistant composite fibers without significative shrinkages [30]. This is the reason why the effect on the acoustical properties of adding silica to electrospun PVP mats was studied. Mats with a high silica content (66%) were successfully electrospun. The electrospinning parameters taken were close to the ones used in our previous work [27] in order to not significantly change the mats structure for which good acoustical properties had been recorded. As a consequence, as will be shown in the paper, the mats structure was much more similar to the pure PVP mats of our previous work [27] than the one reported by Newsome et. al. [30].

The silica particles, 250 nm in diameter, were produced through the Stöber method. Thin disks of Silica/Polyvinylpyrrolidone (PVP) composite were obtained through electrospinning. A second series of disks was obtained by submitting the electrospun mats to proper thermal treatment. The heat treated samples became highly water resistant without suffering shrinkage. Moreover, they passed the 12-s Vertical Bunsen Burner Tests specified in Federal Aviation Regulation (FAR) 25.853 and FAR 25.855 and the smoke density test described in the Aircraft Materials Fire Test Handbook. This is an important point because of severe regulations that often (i.e., in aerospace engineering) prevent materials applicability [31].

The sound absorption coefficients were measured in the same manner as reported in the previous papers [27,28] by using an impedance tube instrument based on ASTM E1050 and ISO 10534-2. The experimental results prove that the large addition of silica (66% of fiber mass) does not change the general features of sound absorption behavior of the mats. Also in the present case, for each set of disks (from a minimum of 6) the sound absorption coefficient changed with the frequency (in the range 200–1600 Hz) following a bell shaped curve with a maximum (where the coefficient is greater than 0.9) that shifts to lower frequencies by increasing the piled disks number. A tunable acoustical response was therefore also obtained in the presence of silica.

This work shows, therefore, that water resistant sound absorbers with reduced thickness and excellent sound-absorption properties in the low and medium frequency range, that pass the fire tests which are mandatory in many engineering areas, may be produced through an eco-friendly electrospinning process. The thermal conductivity was also determined through Differential Scanning Calorimetry (DSC), which allowed us to obtain the thermal conductivity at the melting temperature of Indium (157 °C). The closeness to the value reported for well known thermo-acoustic insulators used in the aerospace engineering (microlite) suggests that the mats also have application potential for thermal insulation.

The experimental results indicate that the good achievements are to be ascribed to the chemical nature of PVP: a polymeric lactam with a side polar group that may be easily released by a thermooxidative process.

## 2. Materials and Methods

### 2.1. Materials

Poly (vinyl pyrrolidone) (PVP) (MW: 1,300,000 g mol^−1^) and tetraethyl orthosilicate (TEOS) reagents were purchased from Sigma Aldrich. Ammonium hydroxide solution (30–33% NH_3_ in H_2_O) and ethanol (99.8% ACS) were purchased from Honeywell.

### 2.2. Methods

#### 2.2.1. Preparation of Electrospun Mats

The silica particles were prepared through Stöber method using tetraethyl orthosilicate (TEOS) as a precursor. TEOS (7 mL) was added to a distilled ethanol (160 mL) water (13.4 mL) and ammonium hydroxide solution (30–33% NH_3_ in H_2_O) (5.38 mL) under stirring. After 2 h the particles were recovered by centrifugation (11500 rpm per 10 min) and washed three times with ethanol.

**Figure 1:**
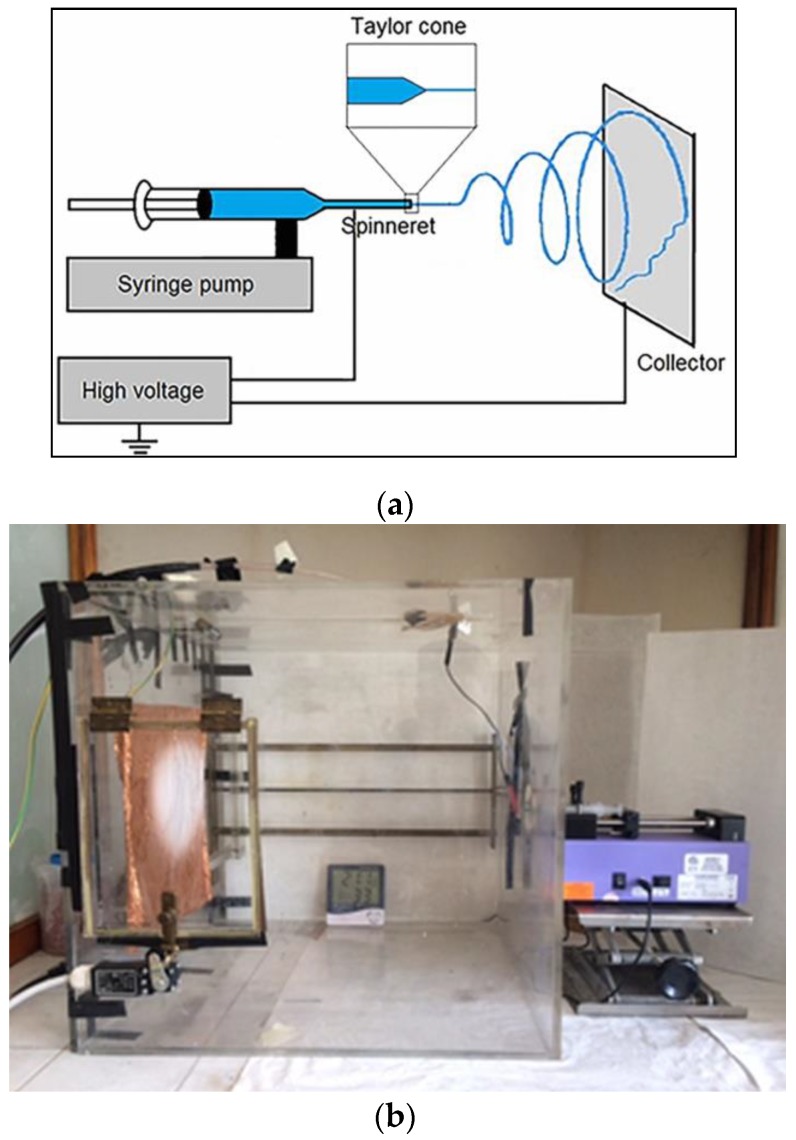
Electrospinning apparatus. (**a**) Schematic setup; (**b**) Laboratory setup.

The scheme of the electrospinning apparatus, shown in Figure 1, consists of a high voltage electric source, a syringe pump (Harvard Apparatus (Cambridge, MA, USA), Pump 11 Plus) holding a 12 mL plastic syringe (Nipro, Osaka, Japan) with a needle (inner diameter of 0.6 mm and average length of 4 cm) acting as the cathode and a rigid copper foil collector acting as the anode.

The electrospun solution was prepared by mixing two other ones:

- An ethanol solution (20 wt. %) of PVP (MW: 1,300,000 g mL^−1^)
- An ethanol suspension (40 wt. %) of silica particles

After 1 h of stirring, the final PVP and silica particles concentrations were 10 and 20 wt. %, respectively.

The PVP/silica particles ethanol suspension was electrospun under a voltage of 30 kV at room temperature and a humidity of 45 ± 10%. A flow rate of 0.100 mL min^−1^ was assured by the syringe pump; the solutions were electrospun over a fixed collection distance of 39 cm. To convey the electrospinning jet onto the collector, a copper string was placed at the bottom of the chamber at the same potential of the needle nozzle. The as-prepared electrospun non-woven mats were dried out at 80 °C for 60 min and stored in a desiccator. The mats were produced in thin sheets corresponding to the content of a syringe.

A second series of samples were submitted to a proper thermal treatment. The electrospun mats were slowly heat treated, in air, from 150 to 200 °C with steps of 10 min each 10 °C. Finally, they were dried out for 6 h at 200 °C.

In the following the two series of samples that were prepared by electrospinning and heat treated, will be distinguished with the acronyms PVP_SiO_2_NT and PVP_SiO_2_HT.

#### 2.2.2. Infrared Spectroscopy (FTIR)

FTIR transmittance spectra were recorded using a Nikolet 5700 FTIR spectrometer (Thermo Fisher, Waltham, MA, USA) using a single reflection Attenuated Total Reflectance (ATR) accessory with a Polymers 2016, 8, 313 5 of 17 resolution of 4 cm^−1^ and 32 scans and Thermo Scientific™ OMNIC™ Software Suite (v7.2, Thermo Fisher, Waltham, MA, USA, 2005).

#### 2.2.3. Scanning Electron Microscopy (SEM)

The morphology of the samples was analyzed by using a field emission scanning electron microscope (FESEM, Zeiss SIGMA) with a nominal resolution of 1.3 nm at 20 kV. SEM images were acquired by collecting secondary electrons (SE) with an Everhart-Thornley (ET-SE) type detector, and by using SmartSEM software (v05.04.00, Carl Zeiss Microscopy GmbH, Jena, Germany, 2016). In some cases, an In-Lens (IL) detector, located inside the electron column of the microscope and arranged rotationally symmetric around the optical axis, was used to collect SEM images with higher contrast.

#### 2.2.4. Thermal Analysis

Thermogravimetric (TGA) analysis was performed using a TA thermogravimetric analyzer (TGA) Q500 V20,13 Build 39. The measurements were conducted in nitrogen, at a heating rate 10 °C min^−1^.

For the thermal conductivity (λ) determination, a Differential Scanning Calorimetry system (DSC Q2000 V24,10 Build 122) equipped with a refrigerated cooling system was employed; TA Universal analysis software (v4.5a, TA Instruments, New Castle, DE, USA, 2018) was used for the data analysis. Tzero Aluminum Hermetic pans were used for these measurements to suppress unwanted signals (e.g., volatile eventually present) and to improve the quality of the analysis. An empty pan of the same type was used as reference in each run. Calibration of the instrument was performed with Indium.

The measurements were carried in a temperature range from −50 °C to 300 °C and the heating rate was 10 °C min^−1^. Four samples of PVP_SiO_2_ HT and an equal number of pure PVP were loaded in the pans. Each pan had a cross sectional diameter of 5.0 mm (cross sectional area A = 0.19625 cm^2^) and a height (L) of 4.0 mm; all the samples were cut into small disks with the same A and different L changing in the range 1–4 mm. The samples preparation was performed according to methodology previously reported [32]. 

#### 2.2.5. Dynamic Light Scattering

Size distribution was measured by software Zetasizer Nano Series (v3.30, Malvern Panalytical, Malvern, Worcestershire, UK, 2008) using a laser dynamic scattering (λ = 632.8 nm) technique.

#### 2.2.6. Fire Test Results

The samples were submitted to two tests required by the Federal Aviation Administration (FAA) in order to assure, in civil aircraft, prescribed levels of fire safety: the Vertical Bunsen Burner Test and the Smoke Density one [33].

#### 2.2.7. Water Resistance

The water resistance was evaluated accordingly to the method proposed in the literature [34], which is shortly described in the following section.

Three randomly selected samples of PVP_SiO_2_HT were placed in a 50 mL beaker in the presence of 30 mL distilled water. After 24 h, the samples were removed and gently rinsed with distilled water.

The weight of water soluble matter, WS, was determined as:

WS = (S° − S)/S°(1)

where S° and S are the weights of the samples before and after immersion in water, both evaluated after drying at 105 °C for 24 h.

#### 2.2.8. Flow Resistivity

As described elsewhere [35] the air flow resistivity was measured according to EN 29053-ISO 9053 through the experimental device SCS 9023 of SCS Controlli e Sistemi s.r.l. and FOAM-X software (v.2007, Mecanum Inc., Sherbrooke, QC, Canada, 2017) was used for data analysis, with the alternate airflow method formerly proposed by Whole and Weber based on a sinusoidal airflow at the frequency of 2 Hz. The reported results are the average of three values obtained on three different samples in the shape of discs of 10 cm diameter and 1 cm thickness.

#### 2.2.9. Sound Absorption Coefficient

The sound absorption coefficient at normal incidence, defined as the ratio between the energy absorbed to the material and the incident energy of the sound wave, indicates the ability of the porous material to absorb sound energy in different frequency bands. It has been measured by means of an acoustic impedance tube in the frequency range 200–1600 Hz according to the geometry of the used instrument (tube diameter and microphones spacing).

**Figure 2:**
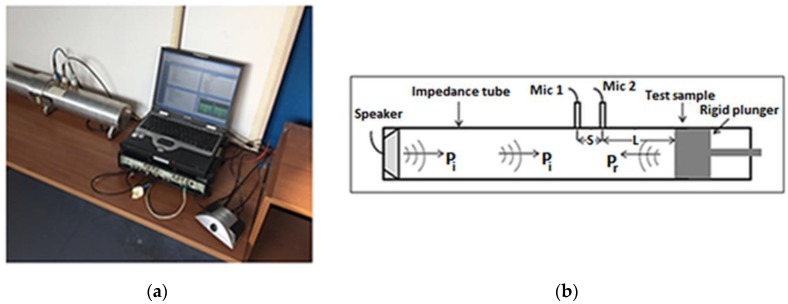
Acoustic sound absorption coefficient measurement. (**a**) Measurement setup in the laboratory; (**b**) Schematic of two-microphone impedance tube method.

The measurement setup consists of an impedance tube (a straight and rigid cylindrical pipe) composed by two main tubes: transmitting and receiving one, a loudspeaker with an amplifier, two microphones and Pulse LabShop software (v6.1.5.65, Brüel&Kjær, Nærum, Denmark, 2002). An image and a sketch of the experimental setup is reported in Figure 2.

The measurements have been performed by employing the two-microphone method (using two microphones in fixed locations) according to ASTM E 1050-12 and ISO 10534-2. The test sample (with a diameter pairs to 100 mm) is mounted at one end of the impedance tube. Plane waves are generated in the transmitted tube by a loudspeaker and the sound pressures are measured at two locations near to the sample.

The complex sound reflection coefficient R of a tested sample is calculated from the corrected acoustic transfer function H_12_. According to Chung and Blaser’s [36] results, the complex sound reflection coefficient is:

R = R_r_ + jR_i_ = ((H_12_ − e^(−jks)^)/(e^jks^ − H_12_))e^(2jk(s + L))^ (2)

where R_r_ and R_i_ are respectively real and imaginary part of complex acoustic reflection coefficient (R), k is wave number, and it is equal to 2πf/c (f is the working frequency, c is the sound speed in the air), L distance from the test sample to the center of the nearest microphone, s center-to-center spacing between microphones, and j = √(−1). From equation (2), it is possible to calculate the sound absorption coefficient at normal incidence as a function of frequency [36,37] and the normal acoustic specific impedance respectively as:

α = 1 − |R|^2^ = 1 − R_r_^2^ − R_i_^2^ (3)

Z/Z_0_ = r/(ρ_0_ c_0_ ) + jx/(ρ_0_ c_0_) = ((1 + R)/(1 − R)) (4)

where Z_0_ = r/(ρ_0_ c_0_) is the characteristic impedance of the medium with p_0_ and c_0_ respectively density and speed of sound in the air, r/(ρ_0_ c_0_ ) is the normal specific acoustic resistance ratio and jx/(ρ_0_ c_0_) is the normal specific acoustic reactance ratio. The normal incidence surface impedance, indeed, is a complex coefficient given by the acoustical pressure to velocity ratio at the surface of the tested sample when it is excited by a normal incidence acoustical wave. It measures the resistance and inertia encountered by the acoustical wave trying to penetrate the material. Its real part is the acoustical resistance and its imaginary part is the acoustical reactance.

**Figure 3:**
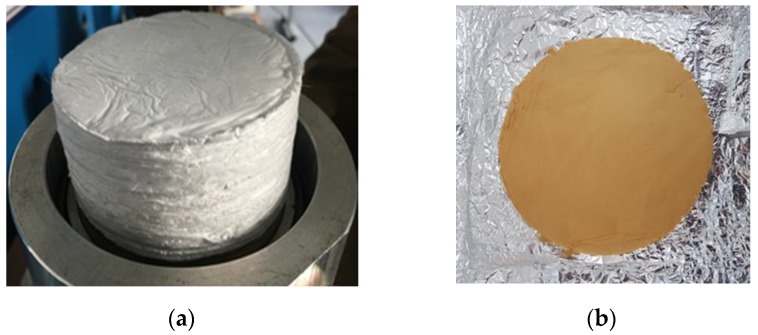
Photographs of electrospun PVP_SiO_2_NT piled mats (**a**) and PVP_SiO_2_HT mat (**b**).

The analysis was made in the same manner so as reported in previous papers [27,28] and shortly reminded in the following. It was performed, at first, on one disk. Then the other disks were added, one by one. After each addition, a pressure was exerted through a metal disk of 130 g for one minute in order to be sure that the disks adhered to each other and to completely eliminate the air layer that could remain entrapped between the electrospun disks. After the measurements, the disks could be easily separated and reassembled in a different order, thus proving that no connection was established between them. The pile of disks was simply leant against the bottom of the acoustic impedance tube. Each measurement was repeated 3–5 times (the test sample was removed and reinserted in the impedance tube each time) and the final result was an average of the repeated measurements. Figure 3 shows the photograph of the final pile of disks prepared for the acoustic impedance tube.

## 3. Results and Discussion

### 3.1. Silica Particles

As is known, the Stöber method is a sol-gel synthesis in alkaline environment [38,39] and is able to give monodisperse sub-micrometric silica gel particles, also a few nanometers in diameter, through the well known hydrolysis and poly-condensation reactions:

Si(OR)_4_ + nH_2_O → Si(OR)_4-n_(OH)_n_ + nROH

≡ Si-OH + HO-Si ≡ → ≡ Si-O-Si ≡ + H_2_O

≡ Si-OH + RO-Si ≡ → ≡ Si-O-Si ≡ + ROH

**Figure 4:**
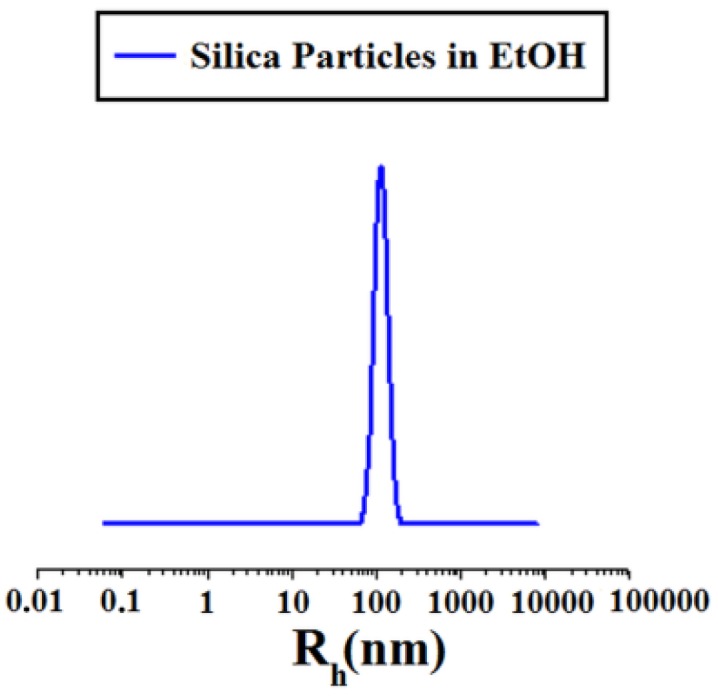
DLS of Stöber particles.

In Figure 4 the DLS (Dynamic Light Scattering) distribution curve of the obtained particles is reported, showing that particles of 140 ± 60 nm radius were successfully obtained.

### 3.2. Electrospun Mats Structure and Composition

**Figure 5:**
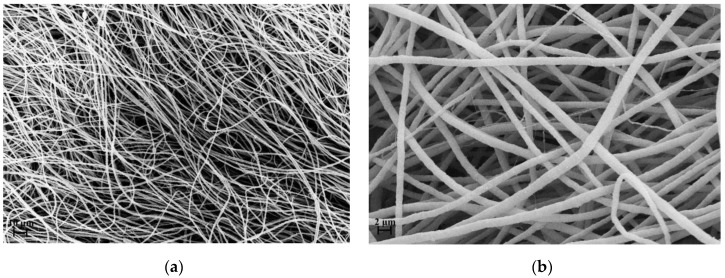
(**a**,**b**) SEM micrographs of not heat treated mat (PVP_SiO_2_NT) at different magnifications.

**Figure 6:**
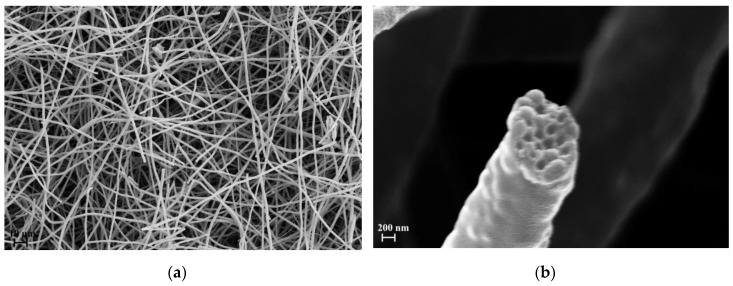
(**a**,**b**) SEM micrograph of heat treated mat (PVP_SiO_2_HT) at different magnifications.

As described in the experimental section, two kind of samples were considered: electrospun that was not heat treated (PVP_SiO_2_NT) and electrospun that was heat treated (PVP_SiO_2_HT). Figure 5 and Figure 6 show the SEM micrographs of both representative samples.

**Figure 7:**
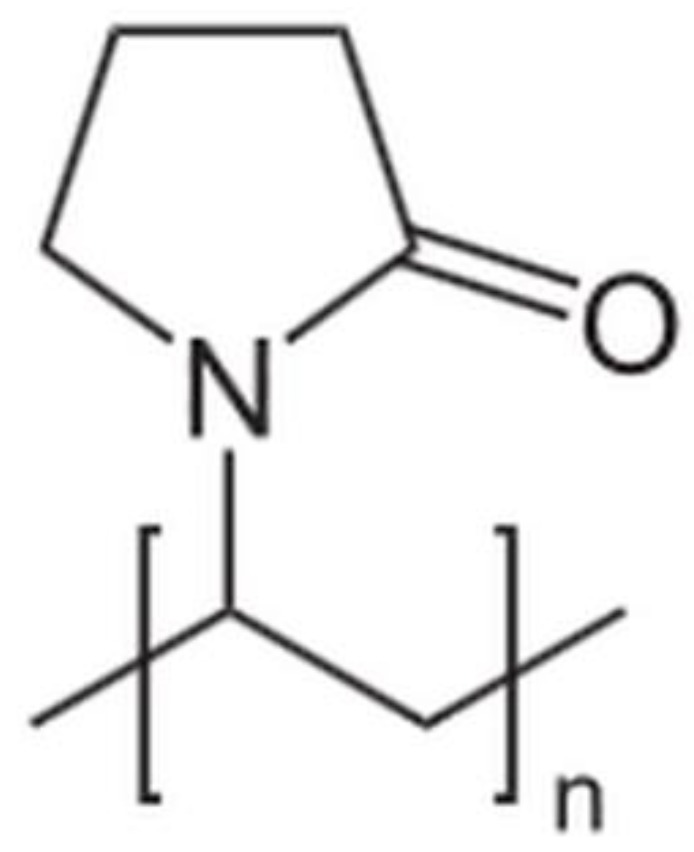
Repetitive unit of PVP.

Figure 5 refers to sample PVP_SiO_2_NT at different magnifications. Analogously Figure 6 refers to sample PVP_SiO_2_HT at different magnifications. The samples do possess the characteristic fibrous structure of electrospun materials. As can be seen, the fibers structure is different than the one reported in the paper of Newsome et al. [30]. This is the consequence of the different electrospinning parameters. Figure 5b shows, in fact, a bimodal distribution of the fiber diameter: 1.6 ± 0.4 μm, close to the one reported in our previous paper for PVP pure mats [27], and 330 ± 30 nm, close to the one reported by Newsome et al. [30]. Figure 6 shows that after thermal treatment a part of the fibers is broken. This allows us to have a glance in the interior of the fibers (see Figure 6b): the particles appear to be tightly arranged inside the fibers separated by thin polymer layers. This may be the consequence of strong absorption interactions between the two phases. It is reported [40,41] that strong hydrogen bonds are expected to form between silanols groups (Si-OH) present at the surface of silica particles and carbonyl groups of PVP whose repetitive unit is reminded in Figure 7.

In Table 1 the fiber diameters are reported altogether with the densities, porosity and flow resistivity of PVP, PVP_SiO_2_NT and PVP_SiO_2_HT blankets. The values for PVP were taken from a previous paper [27].

The values of density, ρ_s_, were estimated with picnometer using hexane. The apparent densities, ρ, were estimated by the volume and weight of a pile of disks like the one photographed in Figure 3. The porosity, that is the ratio of pore volume, V_p_, to bulk volume, V, was calculated with the formula:

P = V_p_/V = 1 − ρ/ρ_s_ (5)

The fiber density, ρ_s,_ doubles on addition of silica. This is clearly due to the greater density of silica with respect to PVP. Although this may lead to a greater apparent density (see value for PVP_SiO_2_NT), a greater porosity and therefore a more open mat structure is obtained when silica is added.

The addition of silica makes the flow resistivity value of sample PVP_SiO_2_NT to increase with respect to the pure PVP mat. This may be the consequence of the presence of the second family of fibers with a diameter 330 ± 30 nm with a much higher specific surface (see Figure 5) which is absent in the pure PVP mat [27]. The decrease in the heat treated sample (PVP_SiO_2_HT) may be tentatively ascribed to the increase of mat looseness linked to the observed fiber breakage (see Figure 6).

**Figure 8:**
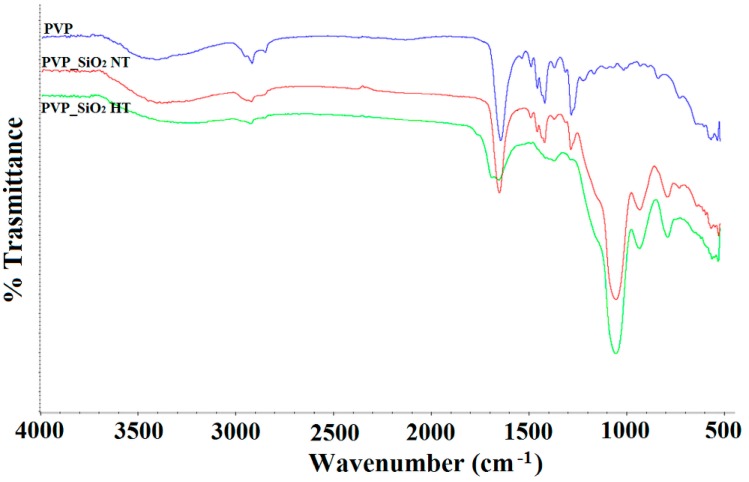
FTIR spectra of PVP_SiO_2_NT (**red line**), PVP_SiO_2_HT (**green line**) and pure PVP (**blue line**).

Figure 8 shows the FTIR spectra of PVP_SiO_2_NT (red line), PVP_SiO_2_HT (green line) and pure PVP (blue line).

In the spectrum of Figure 8 (blue line) the characteristic PVP bands are observed:

- 2930 and 2850 cm^−1^ CH stretching
- 3460 cm^−1^ OH stretching
- 1650 cm^−1^ C = O symmetric stretching
- 1422 cm^−1^ CH_2_ in-plane bending
- 1286 cm^−1^ CN stretching

They are all present in the spectra of Figure 8 (red line) and (green line) altogether with the ones characteristic band of silica (1050 cm^−1^ Si-O-Si stretching).

When comparing Figure 8 (red line) and (green line) a general reduction of intensity of the PVP bands with respect to silica is observed after thermal treatment. A band at 1700 cm^−1^ also appears after the thermal treatment. This band may be attributed to stretching vibration of carbonyl groups in a different environment than the one indicated by the band at 1650 cm^−1^ [40]. It is known in fact [29,42] that thermal degradation of PVP in nitrogen atmosphere occurs at high temperatures, 400–500 °C, with depolymerization of PVP monomer of the polymeric chain. It was also reported, however [40,42], that in oxidative environment, thermal degradation at temperatures over 200 °C, does occur involving partial removal of side pyrrolidone rings and double bond formation along the polymer chain. The band at about 1700 cm^−1^ may be attributed just to stretching of the carbonyl groups conjugated to the double bonds [40] of the polyenic chains that, consequently, form.

Reduction of flexibility due to these cross-links and a brownish color were reported to appear [40]. It’s worth pointing out that our mats consistently acquired a brownish color (see Figure 3) and that a slight mass change (2.54 wt. %) was recorded after the thermal treatment (PVP_SiO_2_HT samples) when the samples were well dried at 100 °C before and after the heat treatment.

**Figure 9:**
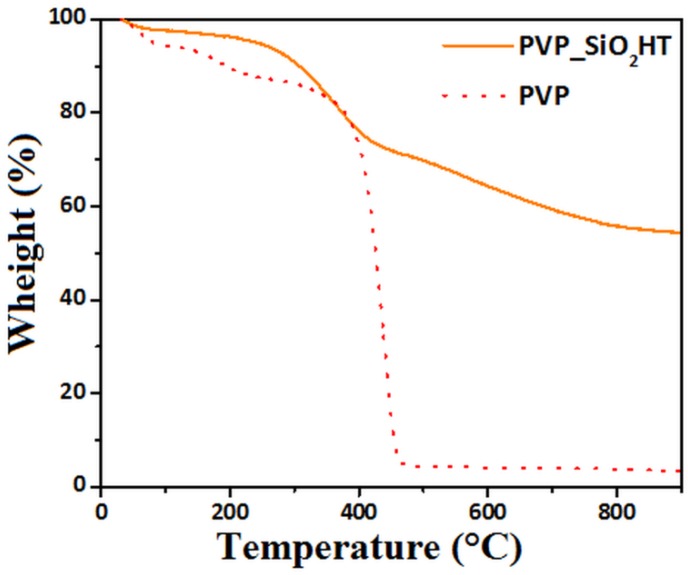
TGA of PVP and PVP_SiO_2_HT.

Figure 9 shows the thermogravimetric curves, recorded in nitrogen atmosphere, of PVP and PVP_SiO_2_HT samples. The PVP shows mass changes below 200 °C that can be attributed to absorbed water release. The greater mass loss shown above 380 °C is, instead, due to its thermal decomposition that is completed at 460 °C. The TGA curve of PVP_SiO_2_HT samples shows the same effects at 460 °C but mass changes are also observed at higher temperatures. These last mass changes may be attributed to the fact that the Stöber method provides gel silica particles. At higher temperatures, volatiles are expected to be released as the consequence of completion of condensation reactions and sintering of the very fine silica gel particles [38,39]. It is worth underlining that the residue at 460 °C is 66% well corresponding to the expected silica content of the fibers on the basis of the composition of electrospun solution reported in Section 2.2.1.

### 3.3. Water Resistance

When submitted to the water resistance test described in the experimental section, the weight percentage change measured on three samples was 1.42 ± 0.50%. The not heat treated sample, instead, rapidly disintegrates when exposed to water. The good water resistance may be attributed to cross-linking occurring during thermal treatment.

### 3.4. Fire Test Results

The sample PVP_SiO_2_HT was submitted to two tests required by the Federal Aviation Administration (FAA) in order to assure, in civil aircraft, prescribed levels of fire safety: the Vertical Bunsen Burner Test and the Smoke Density one [33]. Generally speaking, fire environments to which a material could potentially be exposed are created in order to evaluate the material fire performances.

The Vertical Bunsen Burner Test determines the resistance of materials to flame. A burner flame is applied to a specified specimen for a given ignition time: 12 s or 60 s. Three answers are evaluated:

- Extinguishing time: the time the specimen continues to flame after the burner flame is removed. It is required to be less than 15 s
- Drip extinguishing time: the time, in seconds, that any flaming material continues to flame after falling from the specimen to the floor of the chamber. It is required to be less than 5 s (for the 12 s test)
- Burn length: the distance from the original specimen edge to the farthest evidence of damage to the test specimen. It is required to be less than 203 mm (for the 12 s test)

So as shown in Table 2, the three samples submitted to the test all satisfied the requirements. In particular, no dripping was observed. The video recorded during the test is added in the supplementary materials section (Video S1: Vertical Bunsen Burner Test - PVP_SiO_2_ HT).

The good self-extinguishing properties may be attributed to the structure consisting of tightly assembled silica particles and to the partial thermal degradation that occurred during heating until the temperature reached 200 °C.

The Smoke Density test [33] is a measure of the characteristics of the smoke that may be generated in an airplane passenger cabin. The Specific optical density (Ds)-specific optical density is a dimensionless measure of the amount of smoke produced per unit area by a material when it is burned. This is an important test because smoke excessively dense prevents us from seeing escape routes and may provoke panic. So as shown in Table 3, the medium measured Specific Optical Density was 32.3 much lower than the maximum allowed value of 200.

### 3.5. Thermal Conductivity

**Figure 10:**
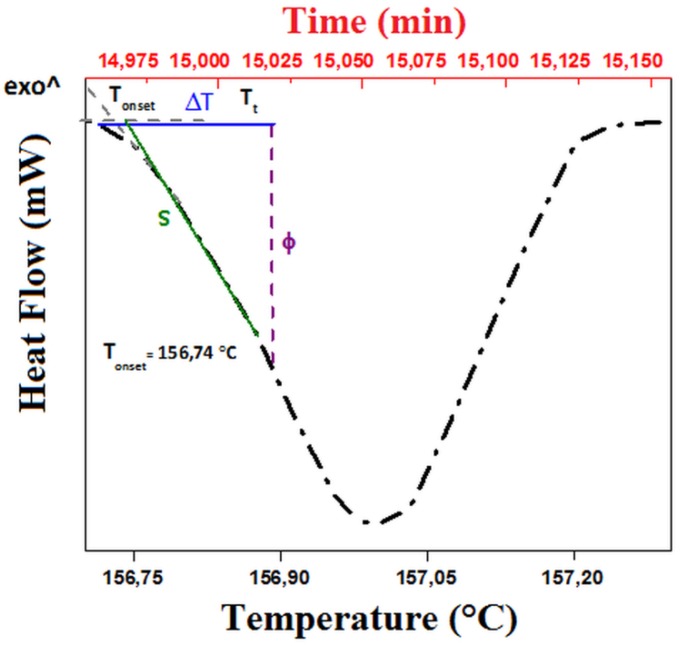
Thermogram recorded in the case of PVP_SiO_2_HT.

Recently several papers proved that thermal conductivity can be measured through a thermal analysis apparatus [32,43]. Some methods use a pure metallic thin disk put on the top of the sample and of its melting to control the temperature of the sample top. Other methods obtain the same control through the addition of a thermal reservoir with temperature sensors. The first ones allow us, of course, to measure the thermal coefficient at the melting temperature of the metal but provide simple and rapid measurements. Thermal conductivity is evaluated from the slope of the endothermic peak appearing when the melting temperature of the metal is reached. Figure 10 shows the thermogram recorded in the present case. The curve shows a characteristic linear trend during melting and decreases after melting is complete, as is reported elsewhere [32]. As can be seen, the onset occurs at the temperature of melting of the metal used in the present case (Indium: melting point 156 °C). The thermal conductance, which is the reverse of thermal resistance, R, can be calculated as the ratio of Φ and ΔT, taken [32] as indicated in Figure 10. If λ, A and L are respectively the thermal conductivity, the section and length of the sample, the thermal resistance is proportional to the ratio L/A: R = L/λA. When the measure is repeated on samples of different ratio L/A, the plot of R as a function of L/A is linear and the thermal conductivity can be measured from its slope [32].

**Figure 11:**
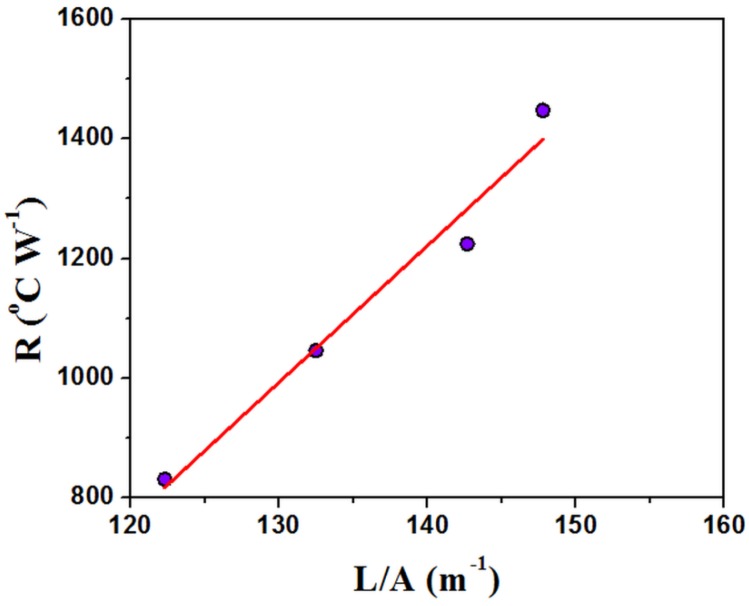
Plot of the thermal resistance, R, as a function of the ratio of the length, L, and section, S of the sample PVP_SiO_2_HT.

As can be seen, a linear plot was obtained in the present case (Figure 11). The analysis gave a value of 0.044 W m^−1^ K^−1^ for the PVP_SiO_2_HT sample, lower than the one for PVP, 0.084 W m^−1^ K^−1^. This value was quite close to the one reported in the literature for pure PVP, 0.12 W m^−1^ K^−1^ [3]. It’s worth pointing out that the value calculated for PVP_SiO_2_HT is close to the one (0.061 W m^−1^ K^−1^ at 149 °C) reported for Microlite^®^AA, a well known Aircraft Acoustical and Thermal Insulation material [44].

### 3.6. Acoustic Properties

**Figure 12:**
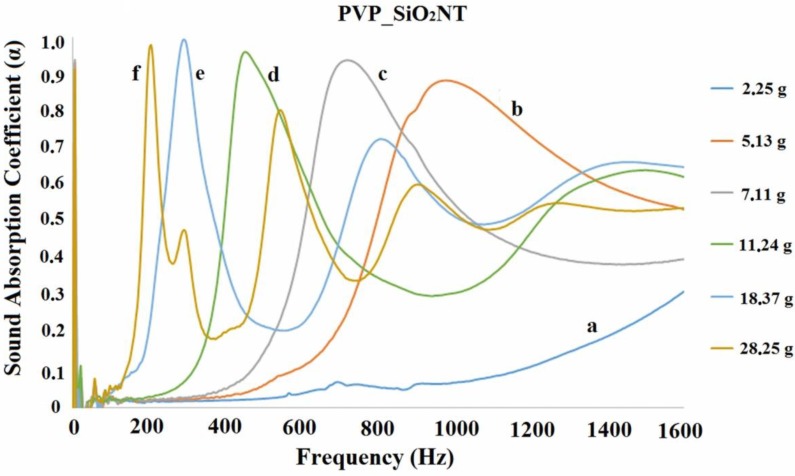
Plots of the sound absorption coefficient as a function of frequency for various PVP_SiO_2_NT disks piles. The a, b, c, d, e and f curves refer, respectively, to samples of total mass 2.25 g, 5.13 g, 7.11 g, 11.24 g, 18.37 g and 28.25 g.

**Figure 13:**
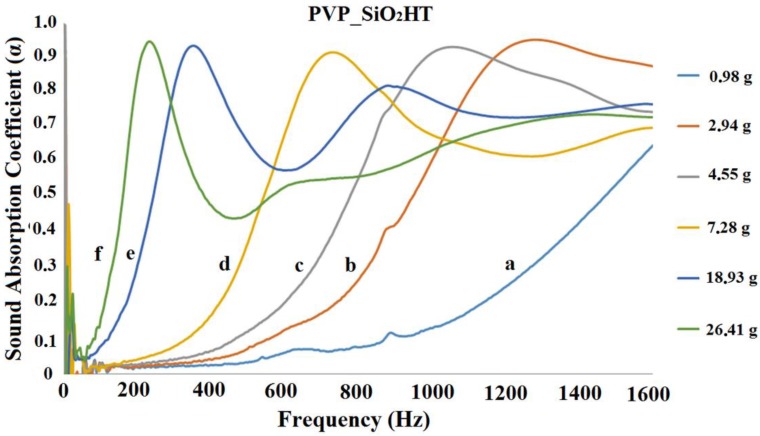
Plots of the sound absorption coefficient as a function of frequency for various PVP_SiO_2_HT disks piles. The a, b, c, d, e and f curves refer, respectively, to samples of total mass 0.98 g, 2.942 g, 4.55 g, 7.28 g, 18.93 g and 26.41 g.

The acoustic impedance tube analysis allowed us to measure the normal incidence sound absorption coefficient in the frequency range 200–1600 Hz, where the performance of traditional porous materials is usually poor. Figure 12 and Figure 13 show some representative results of acoustic impedance tube analysis in the form of the plot of a absorption coefficient, α, as a function of the frequency.

Figure 12 and Figure 13 show that the addition of silica as the successive heat treatment does not change the general trend reported for the pure PVP blankets [27,28]. The absorption coefficient of one disk (curve a) is very low in the examined frequency range. The values of α increase when the disks are stacked on each other. For a total mass of about 5 g (see curve b in Figure 12 and curve c in Figure 13), it appears that the acoustical response of the samples is represented by a bell shaped curve with a maximum α ˃ 0.9. The curves shift towards lower frequency and become sharper by increasing the number (and total mass) of the disks. A second relative maximum was observed for some curves. The second maximum of curves occurred at a frequency of about 3 times greater than the one of the first maximum. In the case of curves d (Figure 12 and Figure 13), the presence of a second maximum may be imagined, even if only the growing part of it is seen. The curves for lower masses might be shifted too much towards higher frequencies to allow for observing the second maximum (beyond 1600 Hz). It is worth underlining that, as previously [27] reported, when the analysis is repeated changing the identity of the disks no differences are observed keeping the total mass constant: the result is thereby related to the total mass, not to the number nor the specific identity of the piled disks.

**Figure 14:**
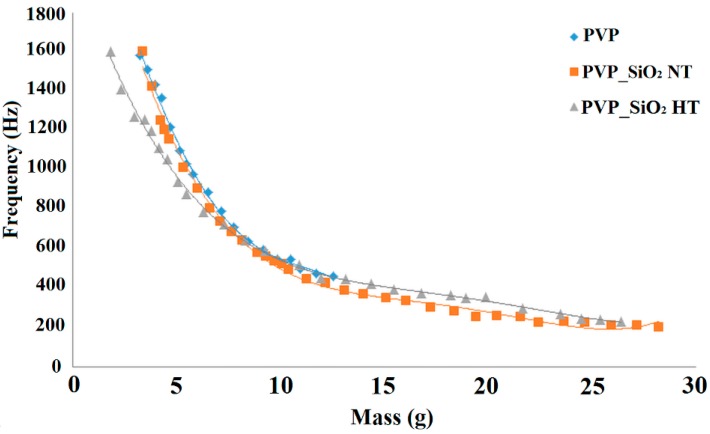
Plots of the frequency of the sound absorption maximum versus the mass per unit surface of the piled disks.

Figure 12 and Figure 13 show only a part of the results. Indeed the measurement was repeated by progressively adding one disk at a time to the pile. The frequency for which α reached the maximum value was determined for each curve. This frequency is reported in Figure 14 as a function of the mass per unit surface of the disks progressively stacked on each other. Figure 14 shows that the shift of the bell like curves towards lower frequencies can be continuously tuned by changing the mass of the blanket.

**Figure 15:**
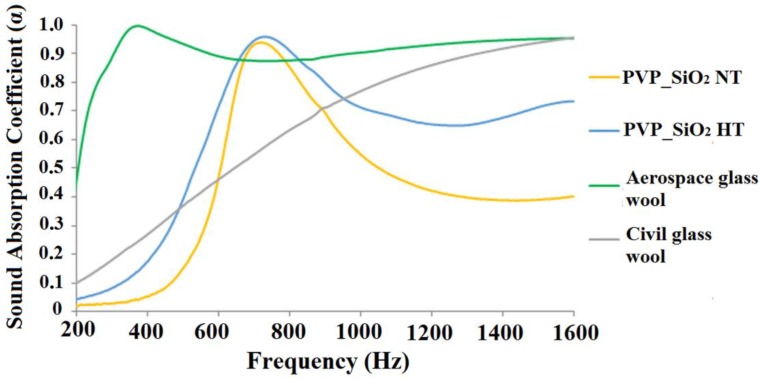
Comparison of the acoustical behaviors of electrospun PVP/silica mats and glass wool samples marketed in civil and aerospace engineering fields of same mass (7.85 g).

**Figure 16:**
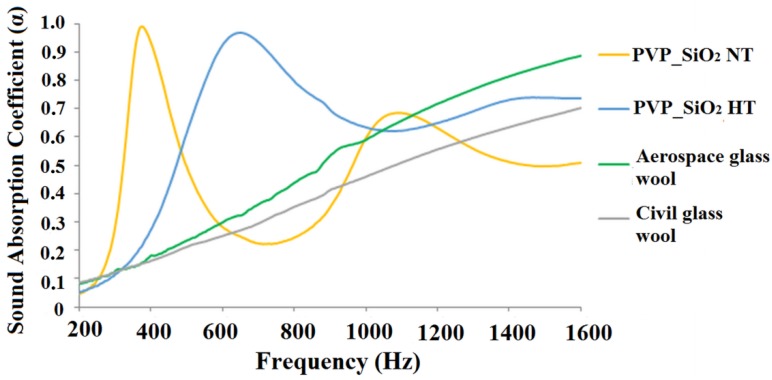
Comparison of the acoustical behaviors of electrospun PVP/silica mats and glass wool samples marketed in civil and aerospace engineering fields of same thickness (2.5 cm).

It is worth underlining a remarkable result: very high sound absorption coefficients were measured. Comparable values of the absorption coefficient, in the 200–1000 Hz frequency range, were reported [26] for heavier and/or thicker samples. This is confirmed by Figure 15 and Figure 16 where the sound absorption curves of electrospun PVP/silica mats and glass wool samples marketed in civil and aerospace engineering fields are compared. In Figure 15, the comparison is made between samples of similar mass. In Figure 16 the results for samples of similar thickness are compared. Figure 15 shows that the electrospun PVP/silica samples may have better sound absorption properties, in the lower frequency range, than materials of the same mass that are usually traded in the field of civil engineering. However, Figure 16 shows that electrospun PVP/silica samples may have better sound absorption properties, in the lower frequency range, than materials, of the same thickness, that are usually used in both the civil and aerospace engineering fields.

## 4. Conclusions

New electrospun PVP/silica mats that preserve the good acoustical properties of pure PVPs, were prepared in the form of thin disks that were piled up. When plotting the acoustical absorption coefficient as a function of frequency, bell shaped curves were recorded, whose maximum (where the coefficient is greater than 0.9) shifts to lower frequencies the greater the mass of piled disks, as already reported by the authors regarding PVP pure mats. The acoustic behavior can be, therefore, continuously tuned by changing the mass of the blanket. They do possess, in the lower frequency range, better sound absorption properties than materials of the same mass that are usually traded in the field of civil engineering and better than materials, of same thickness, usually traded in both civil and aerospace engineering fields.

A thermal treatment was defined that gave mats that, while retaining the good acoustical properties, do possess good water resistance and fire behavior. The structure and composition were studied using SEM, TGA, and FTIR. The density, porosity and flow resistivity were measured.

This paper, therefore, proves that water resistant sound absorbers with reduced thickness and excellent sound-absorption properties in the low and medium frequency range, that are water resistant and pass the fire tests that are mandatory in many engineering areas, may be produced using an eco-friendly electrospinning process. Preliminary investigation of thermal conductivity through Differential Scanning Calorimetry (DSC) shows that they can also be used for thermal insulation.

The experimental results indicate that the achievements can be ascribed to the chemical nature of PVP. It is a polymeric lactam with a side polar group that may be easily released by a thermooxidative process. The side polar groups allow for using ethanol for electrospinning or using a good dispersion of silica gel particles. The silica particles dimensionally stabilize the mats upon thermal treatments that confers water resistance and strongly contribute to the self-extinguishing property of the materials.

## Figures and Tables

**Table 1:** Fiber diameter, densities, porosity and flow resistivity of PVP, PVP_SiO_2_NT and PVP_SiO_2_HT blankets.

	Fiber Diameter [μm]	Apparent Density ρ [g/cm^3^]	Density ρ_s_ [g/cm^3^]	Porosity [%]	Flow Resistivity [kPa (s/m^2^)]
PVP	1.6 ± 0.4	0.063	1.06	94	440
PVP_SiO_2_NT	1.6 ± 0.4 0.33 ± 0.03	0.071	2.08	97	783
PVP_SiO_2_HT	1.6 ± 0.4 0.33 ± 0.03	0.042	2.12	98	289

**Table 2:** Vertical Bunsen Burner Test for Cabin and Cargo Compartment Materials (Federal Aviation Regulation FAR 25.853 and FAR 25.855).

	I T ^2^ (s)	E T ^3^ (s)	Burn Length (mm)	D E T ^4^ (s)	M F T ^5^ (°C)	Coupon Size (cm^3^)	Weight (g)
Sample 1	12	0	57.1	No Drip	843	7.5 × 0.1 × 20	1.3
Sample 2	12	0	70.3	No Drip	843	7.5 × 0.1 × 20	1.2
Sample 3	12	0	50.8	No Drip	843	7.5 × 0.1 × 20	1.1
Results ^1^	/	0	59.4 ± 206	No Drip	/	/	/

^1^ (according to FAR 25.853, FAR 25.855), ^2^ (Ignition Time), ^3^ (Extinguishing Time), ^4^ (Drip Extinguishing Time), ^5^ (Minimum Flame Temperature).

**Table 3:** Smoke Test for Cabin Materials FAR 25.853.

	Specific Optical Density (-)	Coupon Size (cm^3^)	Weight (g)
Sample 1	33.2	7.3 × 0.5 × 7.3	1.1
Sample 2	32.8	7.3 × 0.5 × 7.3	1.2
Sample 3	30.9	7.3 × 0.5 × 7.3	1.3
Results ^1^	32.3 ± 3.02	/	/

^1^ (According to FAR 25.853).

## Supplementary Materials

The following are available online at https://www.mdpi.com/2073-4360/11/7/1205/s1, Video S1: Vertical Bunsen Burner Test - PVP_SiO_2_ HT.
